# Electronic and Magnetic Characterization of Epitaxial CrBr_3_ Monolayers on a Superconducting Substrate

**DOI:** 10.1002/adma.202006850

**Published:** 2021-05-03

**Authors:** Shawulienu Kezilebieke, Orlando J. Silveira, Md N. Huda, Viliam Vaňo, Markus Aapro, Somesh Chandra Ganguli, Jouko Lahtinen, Rhodri Mansell, Sebastiaan van Dijken, Adam S. Foster, Peter Liljeroth

**Affiliations:** ^1^ Department of Applied Physics Aalto University Aalto FI‐00076 Finland; ^2^ Nano Life Science Institute (WPI‐NanoLSI) Kanazawa University Kakuma‐machi Kanazawa 920‐1192 Japan

**Keywords:** chromium tribromide | magnetic 2D materials, niobium diselenide, superconductors, van der Waals heterostructures

## Abstract

The ability to imprint a given material property to another through a proximity effect in layered 2D materials has opened the way to the creation of designer materials. Here, molecular‐beam epitaxy is used for direct synthesis of a superconductor–ferromagnet heterostructure by combining superconducting niobium diselenide (NbSe_2_) with the monolayer ferromagnetic chromium tribromide (CrBr_3_). Using different characterization techniques and density‐functional theory calculations, it is confirmed that the CrBr_3_ monolayer retains its ferromagnetic ordering with a magnetocrystalline anisotropy favoring an out‐of‐plane spin orientation. Low‐temperature scanning tunneling microscopy measurements show a slight reduction of the superconducting gap of NbSe_2_ and the formation of a vortex lattice on the CrBr_3_ layer in experiments under an external magnetic field. The results contribute to the broader framework of exploiting proximity effects to realize novel phenomena in 2D heterostructures.

## Graphical abstract

The electronic and magnetic properties of a 2D monolayer ferromagnet on a layered superconducting substrate are studied using different experimental techniques and theoretical calculations. It is confirmed that the chromium tribromide monolayer retains its ferromagnetic order and induces proximitized magnetism on the underlying superconductor niobium diselenide. The results contribute to the broader framework of exploiting proximity effects to realize novel phenomena in 2D heterostructures.

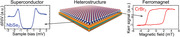

2D magnetic materials constitute an ideal platform to experimentally access the fundamental physics of magnetism in reduced dimensions.^[^ ^1^, ^2^, ^3^ ^]^ Furthermore, because of the ease of fabricating heterostructures, ferromagnetic van der Waals (vdW) materials present attractive opportunities for designer 2D magnetic,^[^ ^4^, ^5^ ^]^ magnetoelectric,^[^ ^6^ ^]^ and magneto‐optical artificial heteromaterials.^[^ ^7^ ^]^ As the different components in a vdW heterstructure only interact via weak vdW forces, the properties of the constituent materials are not strongly modified. This means that one can—for example—imprint magnetic properties of 2D magnets to the other layers without modifying their intrinsic properties and create novel spintronic and magnonic devices.^[^ ^8^, ^9^, ^10^ ^]^ This designer concept can be utilized in systems combining magnetism with superconductivity to realize topological superconductivity.^[^ ^11^, ^12^ ^]^ It is currently attracting intense attention due to its potential role in building blocks for Majorana‐based qubits for topological quantum computation.^[^ ^12^, ^13^, ^14^ ^]^ While there are very few potential real materials exhibiting topological superconductivity,^[^ ^15^, ^16^, ^17^, ^18^ ^]^ in a designer material the desired physics emerges from the engineered interactions between the different components. For topological superconductivity, one needs to combine s‐wave superconductivity with magnetism and spin‐orbit coupling to create an artificial topological superconductor.^[^ ^12^, ^19^ ^]^ However, the coupling between the components is highly sensitive to the interfacial structure and electronic properties^[^ ^2^, ^20^ ^]^ and thus, vdW materials with atomically sharp and highly uniform interfaces are an attractive platform with which to realize and harness exotic electronic phases arising in designer materials.

Layered materials that remain magnetic down to the monolayer (ML) limit have been recently demonstrated.^[^ ^4^, ^5^, ^21^ ^]^ While the first reports relied on mechanical exfoliation for the sample preparation, related materials chromium tribromide (CrBr_3_) and Fe_3_GeTe_2_ have also been grown using molecular‐beam epitaxy (MBE) in ultra‐high vacuum (UHV),^[^ ^22^, ^23^ ^]^ which is essential for realizing clean edges and interfaces. The inherent lack of surface bonding sites due to the layered nature of these materials prevents chemical bonding between the layers and results in a better control of the interfaces. Recently, we have successfully fabricated a superconducting ferromagnetic hybrid system based on vdW heterostructures using MBE.^[^ ^24^, ^25^ ^]^ More importantly, by combining spin‐orbit coupling, 2D ferromagnetic CrBr_3_, and superconducting niobium diselenide (NbSe_2_), we have demonstrated the existence of the 1D Majorana edge modes using low‐temperature scanning tunneling microscopy (STM) and scanning tunneling spectroscopy (STS).^[^ ^25^ ^]^ However, for future applications, further systematic studies are desired for a better understanding of the electronic and magnetic properties of monolayer CrBr_3_ grown on a NbSe_2_ substrate.

In this work, we report a detailed characterization of monolayer CrBr_3_ on NbSe_2_ (CrBr_3_/NbSe_2_ hereafter) using low‐temperature STM/STS and X‐ray photo‐electron spectroscopy (XPS). Combining magneto‐optical Kerr effect (MOKE) measurements and density‐functional theory (DFT) calculations, we unambiguously confirm the ferromagnetism of monolayer CrBr_3_ on a NbSe_2_ substrate. Our results give further experimental information on the magnetic properties of monolayer CrBr_3_, and demonstrate a clean and controllable platform for creating superconducting‐ferromagnetic heterostructure with a great potential for integration into future electronic devices that could be controlled externally through electrical,^[^ ^6^ ^]^ mechanical,^[^ ^26^ ^]^ chemical,^[^ ^27^ ^]^ or optical means.^[^ ^28^ ^]^

In order to study the proximity effects through DFT calculations, we set up a CrBr_3_/NbSe_2_ bilayer heterostructure considering a 2 × 2 supercell for the NbSe_2_ monolayer below a 1 × 1 cell of CrBr_3_ monolayer as shown in **Figure** **1**a, where the 1 × 1 CrBr_3_ consists of Cr atoms arranged in a honeycomb lattice structure and each atom is surrounded by an octahedron of six Br atoms. The energetically most favorable stacking has one Cr atom located on top of a Se_2_ pair, while the other Cr atom is on top of the hole site of the NbSe_2_ (see Experimental Section for details of the calculation). The fully relaxed lattice parameters of the heterostructures reveal that the CrBr_3_ has a tensile strain of about 7 %, while the NbSe_2_ is compressed by less than 2 %, which are not sufficient to drastically affect their electronic and magnetic properties.^[^ ^29^, ^30^ ^]^ XPS spectra for the low binding energy region shown in Figure 1b indicate the presence and stoichiometry of CrBr_3_ on the surface. The Cr 2p spectrum shown in Figure S7 of the Supporting Information confirms that there is only Cr(III) on the surface. Figure 1c shows a large scale STM image illustrating the typical morphology of the monolayer CrBr_3_ grown on NbSe_2_. The monolayer CrBr_3_ islands are atomically flat and up to 200 nm in size, while clean areas of the bare NbSe_2_ substrate are clearly exposed. While the majority of monolayer islands are CrBr_3_, we have consistently observed monolayer CrBr_2_ regions (less than 1% of the CrBr_3_ area) across several STM images and sample preparations as marked with a red arrow in Figure 1c (details given in Figure S4, Supporting Information). Figure 1d shows an atomically resolved STM image of the CrBr_3_ monolayer, revealing periodically spaced triangular protrusions. These features are formed by the three neighboring Br atoms as highlighted in the Figure 1e showing the simulated STM topographic image obtained through DFT calculations. Finally, the STM topography exhibits a clear, well‐ordered superstructure with 6.3 nm periodicity. This superstructure is explained by the fact that when the CrBr_3_ and NbSe_2_ lattices are overlaid, 19 NbSe_2_ unit cells accommodate 10 unit cells of CrBr_3_, thus forming a 6.3 nm × 6.3 nm superstructure (a moiré pattern) as observed in the STM images of CrBr_3_ as shown in Figure 1d. It is important to note that the topographic contrast of the moiré pattern is bias‐dependent (Figure S3, Supporting Information). The rotationally misaligned CrBr_3_ domains have a slight effect on the moiré periodicity (Figure S1, Supporting Information). Additionally, the periodicity of the moiré pattern matches quite well with the lattice parameter of the CrBr_3_ monolayer obtained through DFT (6.37 Å), suggesting that it is most likely unstrained in the CrBr_3_/NbSe_2_ heterostructure.

**Figure 1:**
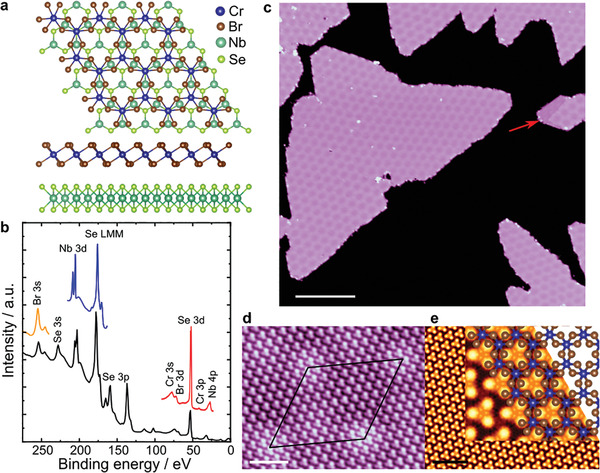
Growth of CrBr_3_ on NbSe_2_. a) Computed top and side views of the CrBr_3_/NbSe_2_ heterostructure. b) Core‐level spectra of CrBr_3_/NbSe_2_ heterostructure. The pass energy was 80 eV for the black curve and 20 eV for colored curves. c) STM image of a monolayer thick CrBr_3_ grown on NbSe_2_ using MBE (STM feedback parameters: *V*_bias_ = +1 V, *I* = 10 pA, scale bar: 39 nm). d) Atomically resolved image on the CrBr_3_ layer. The moiré unit cell is denoted by the large rhombus (STM feedback parameters: *V*_bias_ = +2 V, *I* = 0.5 nA, scale bar: 3 nm). e) Calculated constant‐current STM image of a monolayer of CrBr_3_ on NbSe_2_: *V*_bias_ = +2.0 V , scale bar: 3 nm. The inset in (e) shows a zoom in of the calculated STM image, with the atomic structure of the CrBr_3_ superimposed.

We experimentally determined the electronic structure of the CrBr_3_/NbSe_2_ heterostructure using STS measurements and compared the results directly with DFT calculations. **Figure** **2**a shows a typical STM d*I*/d*V*_b_ spectrum of monolayer CrBr_3_ acquired over a large bias range (a detailed comparison with bare NbSe_2_ spectra is given in the Figure S5, Supporting Information). In the filled state regime (bias voltage *V*_b_ < 0), the spectrum is relatively flat and featureless until *V*_b_ ≈ −1 V is reached, where the d*I*/d*V*_b_ slightly increases, and a steep rise is seen at *V*_b_ ≈ −1.5 V. The dominant feature in the empty state regime (*V*_b_ > 0) of the d*I*/d*V*_b_ spectrum starts with a small bump around ≈0.5 V and a steep rise in d*I*/d*V*_b_ at ≈1.2 V, as shown in Figure 2a. Those features, apart from a rigid shift of ≈0.5 eV, are consistent with the DFT spin polarized projected density of states (PDOS) on the CrBr_3_ layer of the heterostructure shown in Figure 2b. The steep rises in the d*I*/d*V*_b_ spectrum are around 3 V apart and mostly arise from the spin up bands of the CrBr_3_ layer: its electronic properties are well preserved in the heterostructure as compared to the pristine CrBr_3_ layer, as can be seen in Figure 2b,c. The band structures and PDOS shown in Figure 2c reveal that the bands in the [−2.0, −1.0] eV and [−1.0, 1.0] eV windows have a majority contribution from the NbSe_2_ layer, where the NbSe_2_ d‐band in the [−1.0, 1.0] eV window is completely preserved and slightly spin polarized due to the proximity with the magnetic CrBr_3_. However, the PDOS on the CrBr_3_ layer in the [−2.0, −1.0] eV and [−1.0, 1.0] eV windows are nonzero and consistent with the bumps in the d*I*/d*V*_b_ spectrum of Figure 2a. The differential charge density of the CrBr3/NbSe2 heterostructure shown in **Figure** **3**a reveals that charge accumulates at the interface between the layers, as well as around the Cr and Nb atoms, while charge depletes from the Br and Se atoms. Due to this charge reconfiguration close to the CrBr_3_ layer, nonzero states from the Cr and Br atoms are observed within the band gap of the pristine CrBr_3_ layer, which is consistent with the small bumps observed in the d*I*/d*V*_b_ spectra shown in Figure 2a. Such charge reconfiguration is also observed on other similar CrBr_3_/transition metal dichalcogenides (TMDs) and CrI_3_/TMDs heterostructures.^[^ ^31^ ^]^

**Figure 2:**
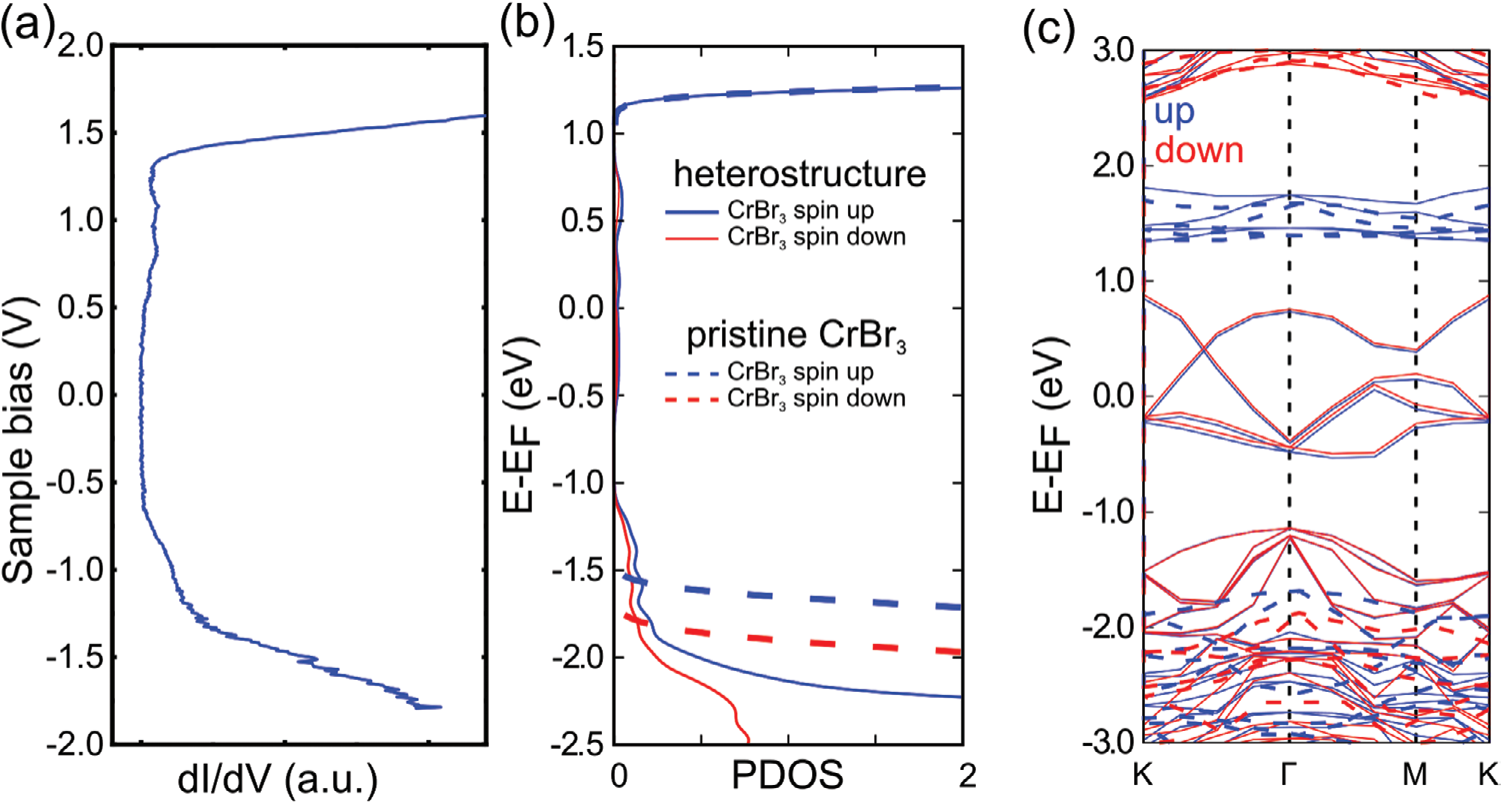
a) Typical long‐range experimental d*I*/d*V*_b_ spectra on a ML CrBr_3_ on the NbSe_2_ substrate. b) Simulated spin polarized projected density of states (PDOS) on the CrBr_3_ layer of the CrBr_3_/NbSe_2_ heterostructure (continuous lines) and on pristine CrBr_3_ layer (dashed lines). c) Band structure of the pristine CrBr_3_ layer (dashed lines) and CrBr_3_/NbSe_2_ heterostructure (continuous lines). The *E*_F_ of the pristine CrBr_3_ was set by aligning its conduction band minimum (CBM) with the correspondent CBM in the heterostructure.

**Figure 3:**
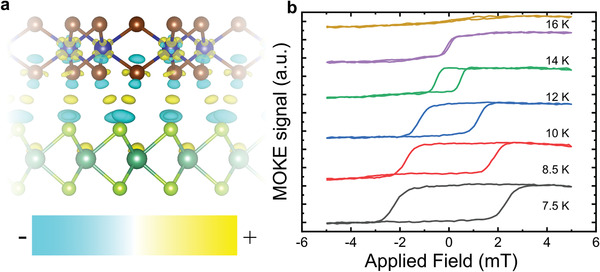
a) Differential charge density of the CrBr_3_/NbSe_2_ heterostructure, where the yellow and blue colors indicate charge accumulation (+) and depletion (−), respectively (isovalue = 3 × 10^−4^ a.u.). b) Magnetic hysteresis measured on the CrBr_3_/NbSe_2_ heterostructure in an out‐of‐plane magnetic field at several different temperatures (indicated in the figure).

After the electronic characterization of the samples, we will next focus on their magnetic properties. The isolated CrBr_3_ layer has a predicted out‐of‐plane magnetic moment of 6.000 μ_B_ per unit cell, where each Cr atom has three unpaired electrons. Our DFT calculations show that the magnetism of the CrBr_3_ layer is well‐preserved in the heterostructure, which shows a slightly larger magnetic moment of 6.097 μ_B_ per unit cell due to induced magnetization on the NbSe_2_ layer. The PDOS on the CrBr_3_ layer of the heterostructure reveals that the majority spin up channel has a band gap of 3 eV, while the minority spin down channel has a band gap of around 5 eV, both shown in blue and red, respectively, in Figure 2c. We study the truly 2D itinerant ferromagnetism in the CrBr_3_ monolayer on NbSe_2_ using magneto‐optical Kerr effect (MOKE) microscopy. The magnetic field was applied perpendicular to the sample using a configuration sensitive to out‐of‐plane magnetization (further details of the experimental procedures are given the Experimental Section). Figure 3b shows the MOKE signal of a CrBr_3_ monolayer on NbSe_2_ as a function of the external magnetic field at several different temperatures. Notably, monolayer CrBr_3_ is ferromagnetic, as evidenced by the prominent hysteresis seen here. As the temperature is increased, the hysteresis loop shrinks and eventually disappears at a transition temperature *T*_c_ of about 16 K. Similar behavior has been observed for a mechanically exfoliated monolayer CrBr_3_ flake, where the transition temperature *T*_c_ was around 27 K.^[^ ^32^ ^]^ Moreover, the out‐of‐plane coercive field *H*_c_ for an exfoliated monolayer CrBr_3_ flake at 5 K has been measured to be ≈8−20 mT^[^ ^28^, ^32^, ^33^ ^]^ while in our MBE grown CrBr_3_ monolayer on a NbSe_2_ substrate the out‐of‐plane coercive field is around 2.5 mT at 7 K. The temperatures stated here are for the cold finger of the cryostat rather than the sample, which may partially explain the discrepancy with previous experiments. Furthermore, our CrBr_3_ layer consists of disconnected, sub‐micron islands, which are significantly smaller than typical exfoliated flakes, which may be expected to reduce *T*_c_. This may also lead to the small coercivity of our layer, although for out‐of‐plane magnetized layers the measured coercivity is strongly dependent on extrinsic factors, which makes comparison with other systems difficult.

After confirming the existence of ferromagnetism in the CrBr_3_/NbSe_2_ heterostructure, it is interesting to see the effects of the superconducting NbSe_2_ substrate on the CrBr_3_ monolayer. Isolated CrBr_3_ is a ferromagnetic insulator; however, due to the monolayer thickness, it is possible to tunnel through such a structure with STM. In addition, due to the charge transfer at the interface between CrBr_3_ and NbSe_2_, the heterostructure itself has a metallic nature. Therefore, one can expect a measurable interaction between CrBr_3_ and NbSe_2_. The superconductivity of the CrBr_3_/NbSe_2_ heterostructure was studied by STM at *T* = 350 mK. **Figures** **4**a,b show experimental d*I*/d*V*_b_ spectra (raw data) taken on a bare NbSe_2_ substrate and on monolayer CrBr_3_, respectively. The d*I*/d*V*_b_ spectrum of bare NbSe_2_ (Figure  4a) has a hard gap with an extended region of zero differential conductance around the Fermi energy. d*I*/d*V*_b_ spectra were fitted by the McMillan two‐band model^[^ ^34^ ^]^ (parameters given in the Supporting Info). In contrast, the spectra taken in the middle of the CrBr_3_ island have small but distinctly nonzero differential conductance inside the gap of the NbSe_2_ substrate. We observe pairs of conductance onsets at ±0.3 mV around zero bias. This feature results from the formation of Shiba‐bands in the NbSe_2_ caused by the induced magnetization from the monolayer CrBr_3_.^[^ ^25^, ^35^ ^]^ These extra features are not reproduced by the two‐band model (Figure 4b, fit parameters given in the Supporting Information).

**Figure 4:**
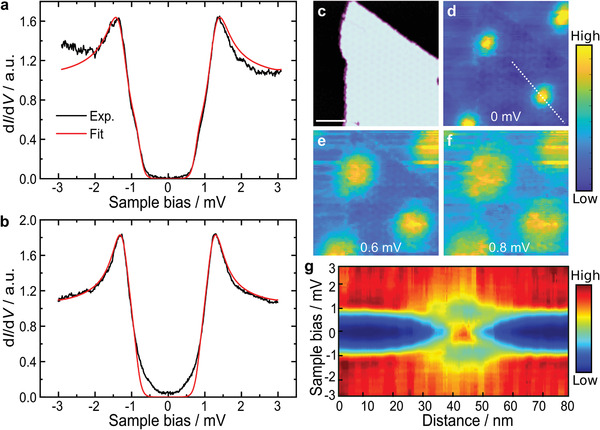
a,b) Experimental d*I*/d*V*_b_ spectroscopy (black) on the NbSe_2_ substrate (a) and in the middle of a CrBr_3_ island (b) measured at *T* = 350 mK. We also show a fit to a double gap s‐wave BCS‐type model (red). c) STM topography image (STM feedback parameters: *V*_bias_ = +1 V, *I* = 10 pA, scale bar: 22 nm). d–f) Vortex imaging on CrBr_3_/NbSe_2_ heterostructure. We have recorded grid spectroscopy (100 × 100 spectra) over an area of 110 × 110 nm^2^ at *T* = 350 mK under an applied out‐of‐plane magnetic field of 0.65 T. The maps are obtained from full spectroscopic scans from −3 to 3 mV at each pixel at the indicated bias voltages. g) Line scan of the d*I*/d*V*_b_ signal along the white line marked in (d).

To obtain more detailed insight into the superconductivity of the CrBr_3_/NbSe_2_ heterostructure, we investigate the dependence of our d*I*/d*V*_b_ spectra under an applied out‐of‐plane magnetic field. In a type‐II SC such as NbSe_2_, we would expect to observe an Abrikosov vortex lattice in a d*I*/d*V*_b_ map acquired near the energy of the superconducting gap.^[^ ^36^ ^]^ Figure 4d–f shows d*I*/d*V*_b_ grid maps at 0, 0.6, and 0.8 mV bias voltages, respectively. The maps are recorded under 0.65 T out‐of‐plane magnetic field on an area with both CrBr_3_ islands and the bare NbSe_2_ surface (Figure 4c). It is seen from Figure 4d–f that the vortices exhibit a highly ordered hexagonal lattice similar to those observed on the clean NbSe_2_ surface. This is the first time vortices have been clearly observed in a hybrid ferromagnet–superconductor‐system. We measured the spatial variation of the d*I*/d*V*_b_ spectra as a function of distance away from the vortex center (along the dashed line in Figure 4d). The results are given in Figure 4g, which shows the measured d*I*/d*V*_b_ as functions of distance and sample bias V on a color scale (spatially resolved d*I*/d*V* *_b_* line spectra of a vortex core state in bare NbSe_2_ are given in Figure S6, Supporting Information). One can see that only one peak appears at zero‐bias in the d*I*/d*V*_b_ spectra near the vortex center, and this peak splits into two away from the vortex core. The splitting energy increases linearly with distance. One of the intriguing properties of a topological superconductor is that vortices on its surface are expected to host Majorana zero modes.^[^ ^37^ ^]^ This mode results in a peak in the local density‐of‐states at the Fermi energy in the center of the vortex. In contrast to bound states in vortices on conventional superconductors, a Majorana mode should not split in energy away from the vortex center.^[^ ^37^, ^38^, ^39^ ^]^ Due to the broadening of the resonance in the vortex spectra, we cannot resolve the individual components in the d*I*/d*V*_b_ spectra and the zero bias feature persists up to 5 nm from the vortex core before the clearly split features can be observed. This is within the range of spatial distributions of the Majorana zero mode reported in the literature (a few nm up to 30 nm).^[^ ^38^, ^39^, ^40^, ^41^ ^]^ In addition, it is important to note that Majorana zero modes in vortex cores have only been observed at low magnetic field (e.g., 0.1 T for Bi_2_Te_3_/NbSe_2_^[^ ^38^, ^41^ ^]^) and its amplitude is expected to decay exponentially as the external field is increased. In order to confirm whether the vortices in our system host Majorana zero modes at their cores, further experiments, in particular using spin‐polarized tunneling measurements,^[^ ^42^ ^]^ are clearly necessary. In summary, we have provided experimental evidence for the realization of a 2D ferromagnet‐superconductor van der Waals heterostructure. More importantly, we have experimentally confirmed that the CrBr_3_ monolayer retains its ferromagnetic ordering with a magnetocrystalline anisotropy favoring an out‐of‐plane spin orientation on NbSe_2_. Our DFT calculations showing an induced moment in Nb from hybridization with Cr d‐orbitals confirm the imprinting of magnetic order on NbSe_2_ from a 2D vdW magnetic insulator. Our results provide a broader framework for employing other proximity effects to tailor materials and realize novel phenomena in 2D heterostructures.

## Experimental Section

### MBE Sample Growth

The CrBr_3_ thin film was grown on a freshly cleaved NbSe_2_ substrate by compound source MBE. The anhydrous CrBr_3_ powder of 99 % purity was evaporated from a Knudsen cell. Before growth, the cell was degassed up to the growth temperature 350°C until the vacuum was better than 1 × 10^−8^ mbar. The sample was heated by electron beam bombardment and temperatures were measured using an optical pyrometer. The growth speed was determined by checking the coverage of the as‐grown samples by STM. The optimal substrate temperature for the growth of CrBr_3_ monolayer films was ≈270°C. Below this temperature, CrBr_3_ forms disordered clusters on the NbSe_2_ surface. The NbSe_2_ crystal is directly mounted on a sample holder using a two component conducting silver epoxy which only allows us to heat the sample up to ≈300°C.

### STM and STS Measurements

After the sample preparation, it was inserted into a low‐temperature STM (Unisoku USM‐1300) housed in the same UHV system and all subsequent experiments were performed at *T* = 350 mK. STM images were taken in constant‐current mode. d*I*/d*V*_b_ spectra were recorded by standard lock‐in detection while sweeping the sample bias in an open feedback loop configuration, with a peak‐to‐peak bias modulation of 30–50 μV for a small bias range and 10 mV for a lager bias range, respectively at a frequency of 707 Hz. Spectra from grid spectroscopy experiments were normalized by the normal state conductance, that is, d*I*/d*V*_b_ at a bias voltage corresponding to a few times the superconducting gap.

### Sample Transfer

After sample measurement in STM, the sample was transferred to the load lock (≈10^−9^ mbar). We connected a glove bag directly to the load lock and vented it slowly with pure nitrogen. The sample was then transferred via the glove bag for XPS and MOKE measurements.

### XPS Experiments

The XPS spectra were measured using a Kratos Axis Ultra system, equipped with monochromatic Al K_α_ X‐ray source. All measurements were performed using an analysis area of 0.3 mm × 0.7 mm. The black curve in Figure 1b was measured using 80 eV pass energy and 1 eV energy step whereas the colored curves were taken with 20 eV pass energy and 0.1 eV energy step. The energy calibration was done using the C 1s peak at 284.8 eV.

### MOKE Measurements

MOKE was carried out using an Evico Magnetics system based on a Zeiss Axio Imager D1 microscope with a Hamamatsu C4742‐95 digital camera. The sample was placed in a Janis research ST‐500 cold finger cryostat with optical access and cooled using liquid He. Imaging of the sample was carried out in a polar Kerr configuration under an out‐of‐plane magnetic field. Hysteresis loops were constructed from images taken using a long working distance 100× lens after background image subtraction and corrected for a linear background slope due to the Faraday effect on the lens.

### DFT Calculations

Calculations were performed with the DFT methodology as implemented in the periodic plane‐wave basis VASP code.^[^ ^43^, ^44^ ^]^ Atomic positions and lattice parameters were obtained by fully relaxing all structures using the spin‐polarized Perdew–Burke–Ernzehof functional^[^ ^45^ ^]^ including Grimme's semiempirical DFT‐D3 scheme for dispersion correction,^[^ ^46^ ^]^ which is important to describe the vdW interactions between the CrBr_3_ and the NbSe_2_ layers. The stacking of the layers used in the calculations is the same used in a previous work.^[^ ^25^ ^]^ The convergence criterion of self‐consistent field computation was set to 10^−5^ eV and the threshold for the largest force acting on the atoms was set to less than 0.012 eV Å^−1^. A vacuum layer of 12 Å was added to avoid mirror interactions between periodic images. Further calculations of band structures and density of states were realized using the hybrid Heyd–Scuseria–Ernzerhof (HSE06) functional,^[^ ^47^, ^48^, ^49^ ^]^ which improves the description of the band structure as compared to the PBE functional. The interactions between electrons and ions were described by PAW pseudopotentials, where 4s and 4p shells were added explicitly as semicore states for Nb and 3p shells for Cr. An energy cutoff of 550 eV was used to expand the wave functions and a systematic *k*‐point convergence was checked, where the total energy was converged to the order of 10^−4^ eV. Spin polarization was considered by setting an initial out‐of‐plane magnetization of 3 μ_B_ per Cr atom and zero otherwise.

## Conflict of Interest

The authors declare no conflict of interest.

## Supporting information

Supporting Information
