# Supplementary material for: Electronic and Magnetic Characterization of Epitaxial CrBr3 Monolayers on a Superconducting Substrate
Source: Adv Mater. 2021 May 3;33(23):2006850. doi: 10.1002/adma.202006850 (PMC11468557; doi:10.1002/adma.202006850)
Supplement: Supplementary file 1 — Supporting Information [file ADMA-33-2006850-s001.pdf]

© 2021 Wiley-VCH GmbH

ADVANCED MATERIALS

Supporting Information

for *Adv. Mater.,* DOI: 10.1002/adma.202006850

Electronic and Magnetic Characterization of Epitaxial $CrBr_3$ Monolayers on a Superconducting Substrate

Shawulienu Kezilebieke, Orlando J. Silveira, Md N. Huda, Viliam Vaňo, Markus Aapro, Somesh Chandra Ganguli, Jouko Lahtinen, Rhodri Mansell, Sebastiaanvan Dijken, Adam S. Foster, and Peter Liljeroth*

Supporting Information: Electronic and magnetic characterization of epitaxial $CrBr_3$ monolayers

Shawulienu Kezilebieke [a],[1,*] Orlando J. Silveira [a],[1] Md N. Huda [a],[1] Viliam Vaňo,[1] Markus Aapro,[1] Somesh C. Ganguli,[1] Jouko Lahtinen,[1] Rhodri Mansell,[1] Sebastiaan van Dijken,[1] Adam S. Foster,[1,2] and Peter Liljeroth[1]

[1]*Department of Applied Physics, Aalto University, FI-00076 Aalto, Finland*

[2]*Nano Life Science Institute (WPI-NanoLSI), Kanazawa University, Kakuma-machi, Kanazawa 920-1192, Japan*

1. MOIRÉ PATTERN ANALYSIS

The periodicities and relative angles of the moiré pattern were measured from the Fourier spots of plane-subtracted constant current STM scans. The (fast) Fourier transform preserves the relative periodicities and angles between the lattices regardless of the normalisation convention used. The scans used in the analysis were taken with various feedback parameter combinations: although the pattern intensity evolves with bias voltage, we did not observe noticeable bias dependence in the moiré periodicities and angles. The peak positions were fitted manually using Gwyddion [1].

Figure 2 (c) shows the parameters measured for 27 atomic resolution scans of the moiré pattern. Limited k-space resolution introduces variance especially in the angle data. It should be noted that no methods to remove the effects of piezo drift were used in the analysis: the effects of drift were assumed negligible due to reasonably fast scan speeds (~ 30 nm/s).

[a] These authors contributed equally.

[*] shawulienu.kezilebieke@aalto.fi

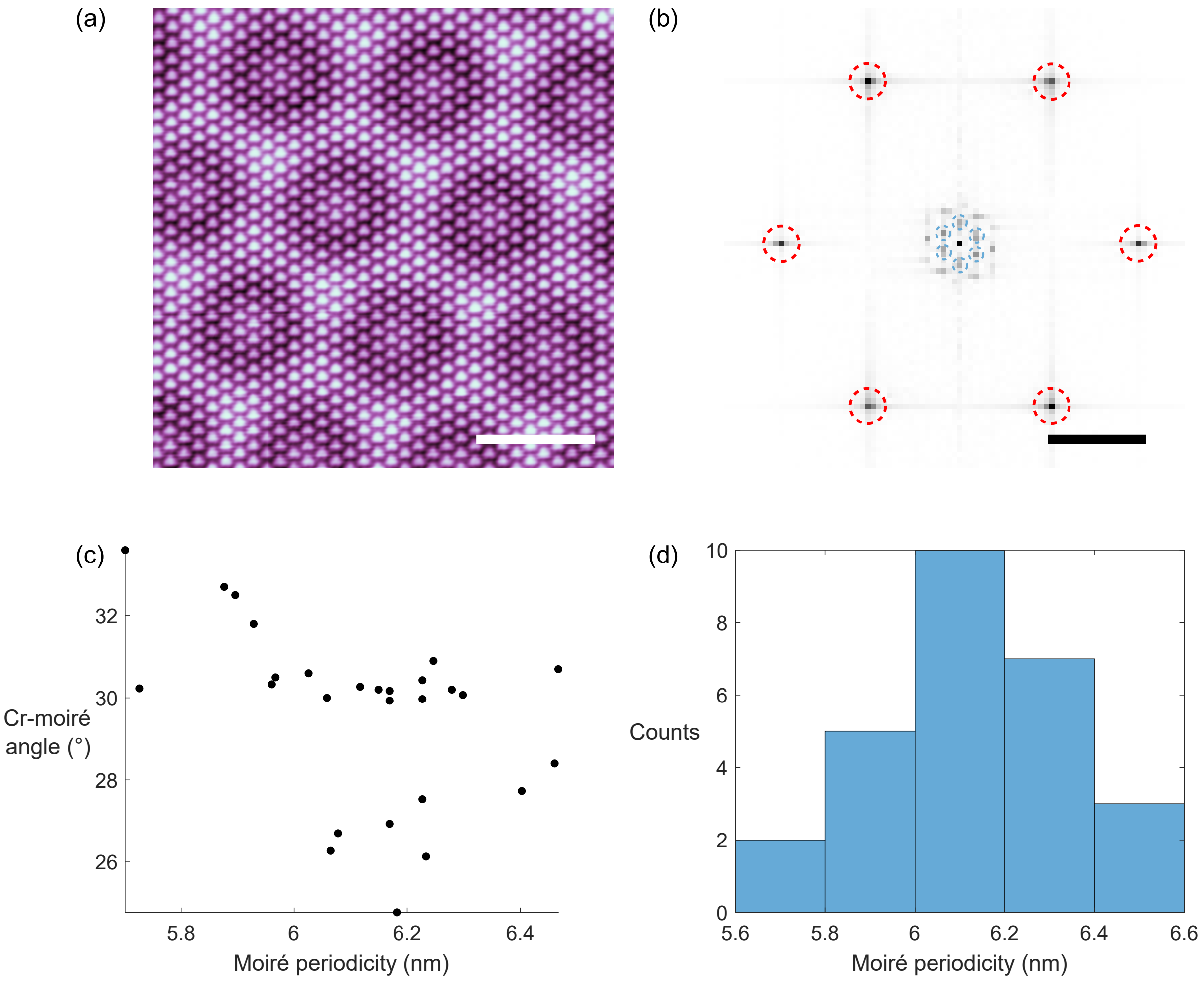

Supplementary Figure 1. Moiré pattern analysis. (a) Constant current scan of the moiré pattern at atomic resolution (STM feedback parameters: $V_{\text{bias}} = +2$ V, $I = 500$ pA, scale bar: 5 nm) (b) Fast Fourier transform of (a). Chromium lattice and moire spots are highlighted with red and blue circles, respectively. Scale bar 1 nm^{-1}. (c) Scatter plot of the measured moiré pattern periodicities and angles with respect to the chromium lattice, $N = 27$. (d) Histogram of moiré periodicity.

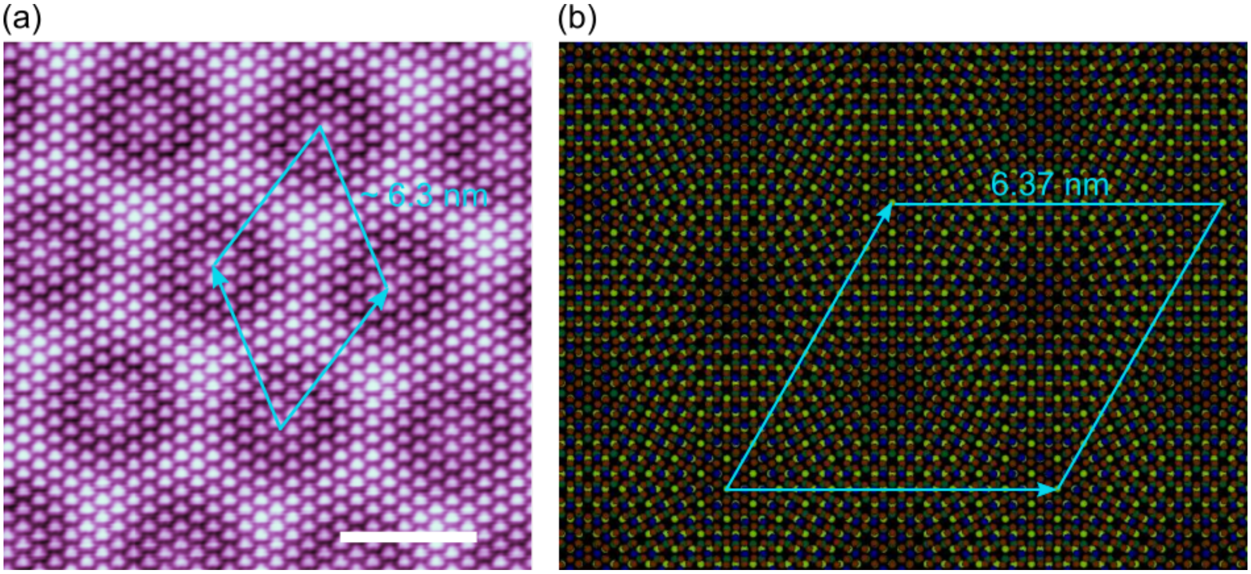

Supplementary Figure 2. (a) Constant current scan of the moiré pattern at atomic resolution (STM feedback parameters: V_{bias}= +2 V, I= 500 pA, scale bar: 5 nm. (b) Schematic representation of the moiré pattern with a periodicity of 6.328 nm. The moiré pattern was obtained using the geometries of the isolated compounds $CrBr_3$ and $NbSe_2$ obtained through DFT calculations and brought together considering a twisting angle of 30°.

2. BIAS-DEPENDENT STM IMAGES OF THE MOIRÉ PATTERN

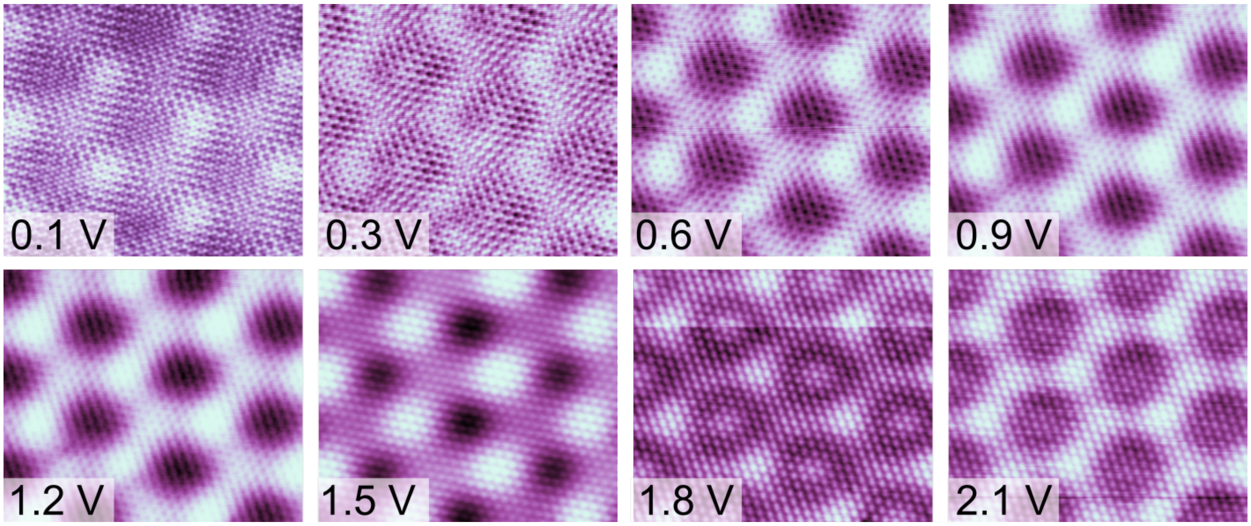

Supplementary Figure 3. Bias-dependent STM images of the $CrBr_3$ moiré pattern with the bias voltage indicated in each panel.Image size 20×17 nm^2

3. STM IMAGE OF CRBR$_2$

We have also observed (less than 1% of the $CrBr_3$ area) a ”special” type of regions as shown in the Fig.1 in the main text across several STM images and sample preparations. Zoomed in STM image of such a region is shown in the Figure 4a. First of all, the height of those “special regions” is 0.2 Å smaller than the normal $CrBr_3$ island height. Secondly, the lattice constant of the “special region” is ca 3.9 Å(Figure 4b,c), which is consistent with that of $CrBr_2$ (reported to be 3.7 Å, [2]). Therefore, we think these regions are $CrBr_2$ caused by the decomposition and loss of Br during the growth of $CrBr_3$ by sublimation.

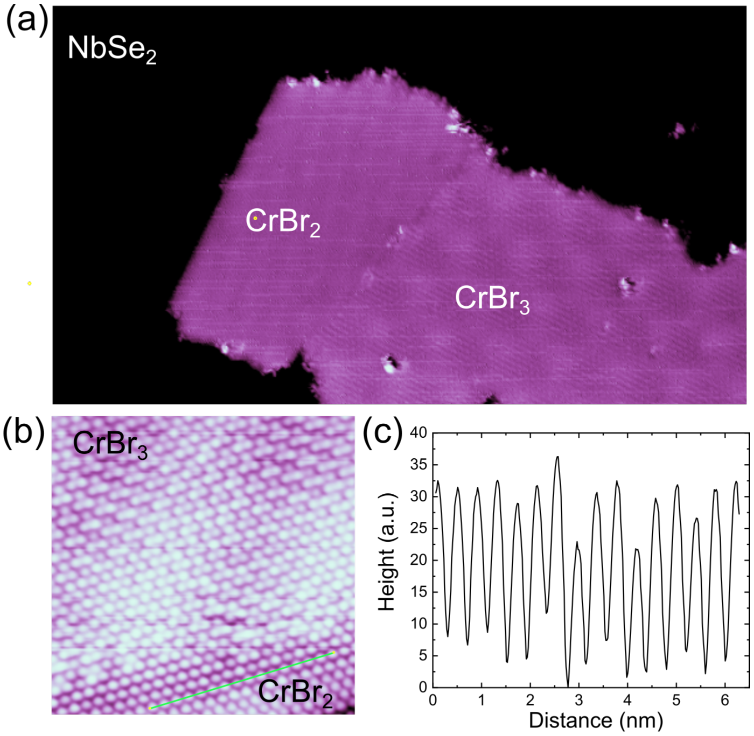

Supplementary Figure 4. (a) STM image of submonolayer $CrBr_2$ and $CrBr_3$ on $NbSe_2$. Image size 75×43 nm^2. (b) Atomically resolved image of another $CrBr_3$ / $CrBr_2$ interface. Image size 10×10 nm^2. (c) Line profiles along the line in panel (b) showing the lattice constant of the $CrBr_2$ to be ca. 3.9 Å.

4. A LARGE BIAS RANGE DI/DV SPECTRA

Figure 5 shows the results of careful spectroscopy experiments focusing on the position

of the spectroscopic features arising from the Nb d-band. Figure 5a,b shows large bias range overview spectra. While the measurement on bare $NbSe_2$ shows the d-band close to zero bias, the spectrum on the $CrBr_3$ island only shows the conduction band onset (at a bias of 1.2 V) and some very faint features at smaller bias. However, focusing on biases closer to zero and increasing the setpoint current sufficiently, allows also the resolution of the Nbd-band features under the $CrBr_3$. This is shown in Figure 5c, where $NbSe_2$ d-band is completely preserved. Moreover, $NbSe_2$ d-band is shifted slightly towards higher energy under $CrBr_3$ by ca 80 mV.

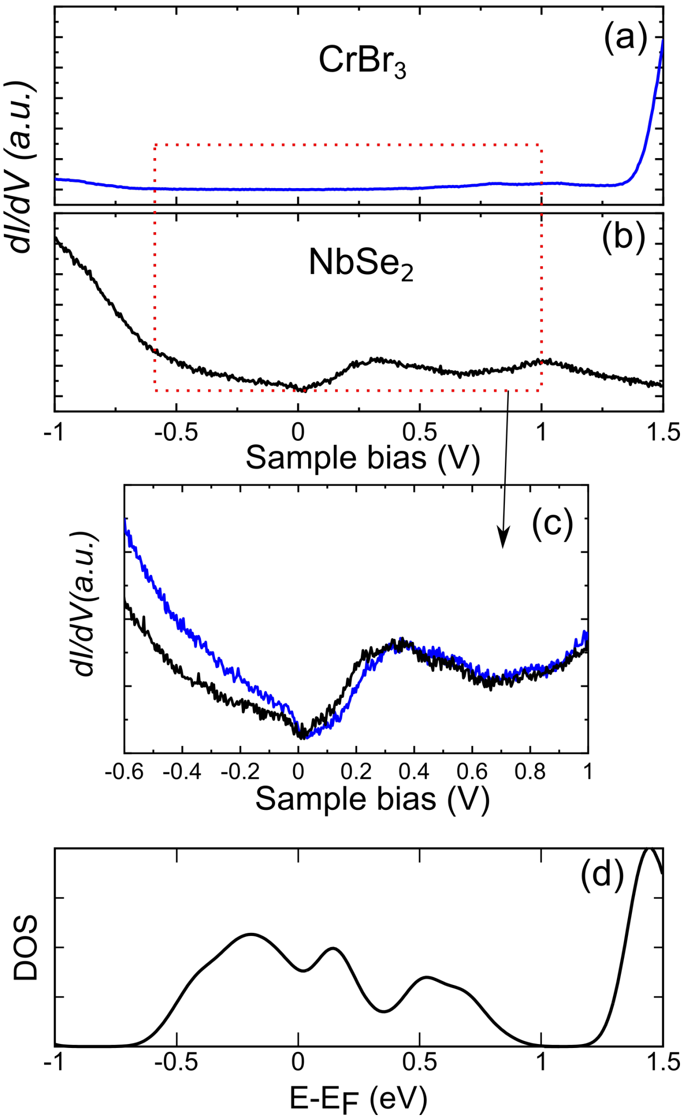

Supplementary Figure 5. Comparison of $\mathrm{d}I/\mathrm{d}V$ spectra on bare $NbSe_2$ and on $CrBr_3$. (a) Large bias range spectra on $CrBr_3$. (b) Large bias range spectra on $NbSe_2$. (c) Spectroscopy within the gap of the $CrBr_3$ on $NbSe_2$ (blue line) and on $NbSe_2$ (black line). (d) Density of states (DOS) of the $CrBr_3/NbSe_2$ heterostructure.

5. MCMILLAN TWO-BAND MODEL

TABLE I. Best-fit parameters of the dI/dV_b spectra to the McMillan two-band model on clean $NbSe_2$ and $CrBr_3/NbSe_2$ samples.

sample	Δ_1 (meV)	γ_1 (meV)	Δ_2 (meV)	γ_2 (meV)
clean $NbSe_2$	1.28	0.38	0.74	0.13
$CrBr_3/NbSe_2$	1.20	0.40	0.73	0.50

6. SPATIALLY RESOLVED $NBSE_2$ VORTEX CORE STATES

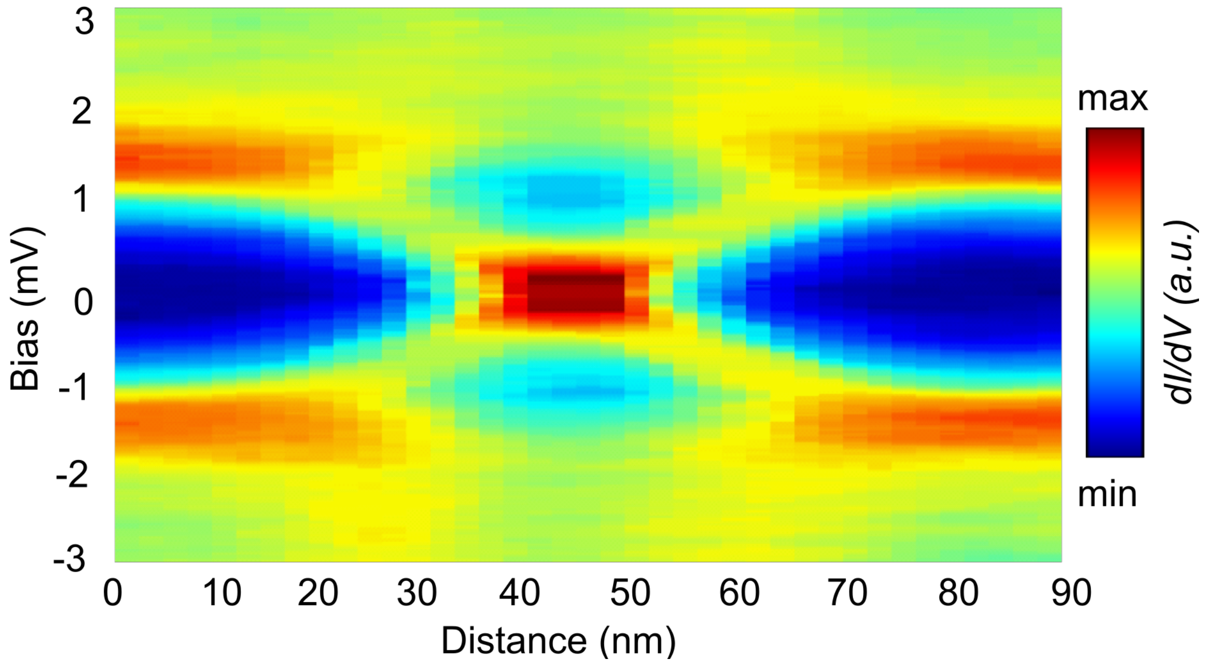

Supplementary Figure 6. dI/dV line spectra measured across a single vortex on a bare $NbSe_2$.

7. X-RAY PHOTOELECTRON SPECTROSCOPY

The XPS spectrum of the Cr 2p region is shown in (Figure 7 and the shape clearly indicates a single oxidation state. The solid line represents a fit with one GL(30) peak

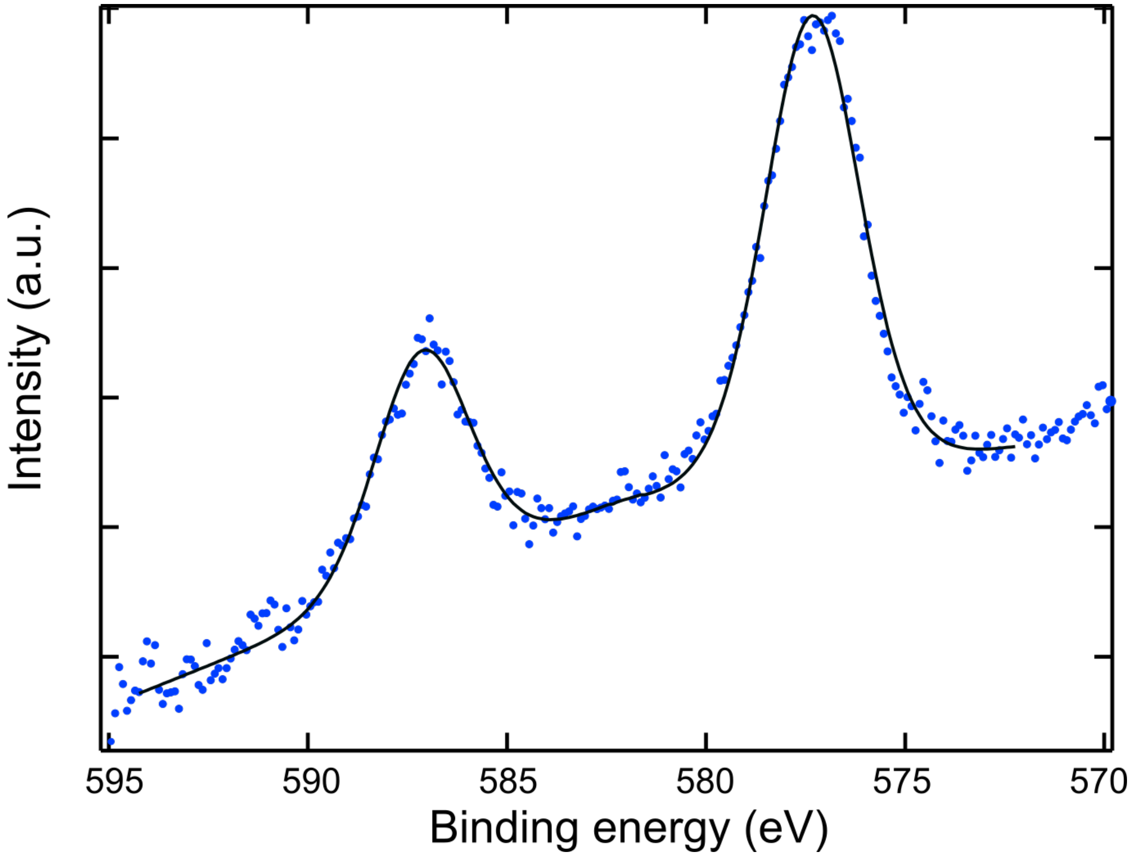

Supplementary Figure 7. Cr 2p XPS spectrum. The solid line represents fitting with single GL(30) peak for both components.

(Product of 30% Lorenzian and 70% Gaussian) for both $p_{3/2}$ and $p_{1/2}$. The Cr 2$p_{3/2}$ peak appeared at 577.3 eV, the doublet separation was 9.8 eV and the peak width 2.8 eV. These values match with the approximate values obtained for $CrBr_3$ [3, 4]. The binding energy is typical for Cr(III) as the same value has been reported also for Cr(III)hydroxide with a similar peak width [5].The binding energy reference was the C 1s at 284.8 eV.

---

[1] D. Nečas, P. Klapetek, *Cent. Eur. J. Phys.* **2012**, *10*, 181–188.

[2] V. V. Kulish, W. Huang, *J. Mater. Chem. C* **2017**, *5*, 8734–8741.

[3] I. Pollini, *Phys. Status Solidi B* **2000**, *222*, 483–493.

[4] J. C. Carver, G. K. Schweitzer, T. A. Carlson, *J. Chem. Phys.* **2020**, *57*, 973–982.

[5] M. C. Biesinger, B. P. Payne, A. P. Grosvenor, L. W. M. Lau, A. R. Gerson, R. S. C. Smart, *Appl. Surf. Sci.* **2011**, *257*, 2717–2730.
